# Cord-derived mesenchymal stem cells therapy for liver cirrhosis in children with refractory Henoch–Schonlein purpura: A case report

**DOI:** 10.1097/MD.0000000000013287

**Published:** 2018-11-21

**Authors:** Kai Mu, Jing Zhang, Yan Gu, Hongjuan Li, Yan Han, Na Cheng, Xiaoyu Feng, Guoyu Ding, Rongjun Zhang, Yuqi Zhao, Hongmei Wang

**Affiliations:** Department of Pediatrics, Shandong Provincial Qianfoshan Hospital of Shandong University, Jinan, China.

**Keywords:** child, cord-derived mesenchymal stem cells, Henoch–Schonlein purpura, liver cirrhosis

## Abstract

**Rationale:**

To explore the curative effect of human umbilical cord-derived mesenchymal stem cell (ucMSC) therapy for patients with liver cirrhosis complicated with immune thrombocytopenia and refractory Henoch–Schonlein purpura (HSP).

**Patient concerns:**

A 12-year-old boy presented to our hospital with an 11-month history of purpura on the skin of both lower limbs accompanied by thrombocytopenia. The patient had a history of repeated swelling and painful dorsum pedis, followed by skin redness.

**Diagnosis:**

Bone marrow slides showed megakaryocyte maturation disorder. Based on the pathology and drug abuse history, he was diagnosed with nodular cirrhosis, secondary allergic purpura, and thrombocytopenia, etiologies related to his drugs and an immune dysfunction.

**Interventions:**

ucMSC transplantation was performed, the liver damaging drugs were discontinued, and the appropriate liver immunosuppressive drugs were administered. ucMSCs were injected 8 times/wk in 2 months, with a median cell count of 5.65 × 10^7^/L, ranging from 5.48 to 5.98 × 10^7^/L.

**Outcomes:**

As the patient's skin rash resolved, his platelets gradually increased to >150 × 10^9^/L and liver transaminase levels gradually decreased to a normal level. Ultrasonography of the abdomen indicated that the round nodules in the liver decreased in size and that the spleen thickness also decreased.

**Lessons:**

This is a unique case of significant HSP with associated thrombocytopenia in a patient with liver cirrhosis. Long-term oral administration of excessive herbal medicine may cause liver damage. We believe that ucMSCs provide a novel approach for the treatment of liver cirrhosis.

## 1 Introduction

Henoch–Schonlein purpura (HSP) is a 1-vessel systemic vasculitis caused by vascular allergic inflammation, and predominantly affects children.^[1]^ Idiopathic thrombocytopenia (ITP) is an acquired autoimmune disease characterized by low platelet count and the presence or absence of skin and mucous membrane bleeding. Umbilical cord mesenchymal stem cells (ucMSCs) are not only obtained by a noninvasive procedure, but can also be cultured relatively easily, which makes them potentially superior to MSCs from other sources for cell transplantation therapy.^[2]^ Some articles revealed that ucMSCs transplanted into acutely injured and fibrotic livers could restore liver function and improve liver fibrosis.^[3,4]^ The present study explores the curative effect of human ucMSC therapy for patients with liver cirrhosis complicated with immune thrombocytopenia and refractory HSP. Since this condition is rare, the insights obtained by this study would be helpful to other researchers and clinicians.

## 2 Case report

Informed consent was obtained from the patient for the publication of this case report and its accompanying images. A 12-year-old boy visited our hospital with symptoms of purpura in the skin of the bilateral lower limbs, accompanied by thrombocytopenia. The patient had a history of a chronic itching skin rash for 2 years. He was treated with oral drugs including prednisone, vitamin C, *Tripterygium wilfordii*, and other traditional Chinese medicines. Beginning 11 months prior, the patient developed repeated episodes of swelling, a painful dorsum pedis, followed by erythema (that did not fade with pressure), and a low platelet count (58 × 10^9^/L). He was diagnosed with HSP with thrombocytopenia in a local hospital and received symptomatic treatment, such as prednisone, vitamin C, and calcium carbonate. The platelet count remained very low (26 × 10^9^/L). A megakaryocyte maturation disorder and positive identification of glycoprotein antibodies (GP IIb) were confirmed in the patient's bone marrow smear. These findings confirmed the diagnosis of immune thrombocytopenia. In another hospital, after the patient was administered caffeic acid, a compound soap alum pill, and other oral drug preparations (approximately 10 oral drugs), the platelet count increased to a normal range. Approximately 2 weeks before admission to our hospital, the patient developed dorsal foot pain and purpura on the skin of both lower limbs. After oral application of traditional Chinese medicine and vitamin C for 1 week, the purpura reduced slightly. One week later, the patient experienced sudden paroxysmal abdominal pain with more intense purpura and skin itching. The platelet count at that time was 135 × 10^9^/L. Food allergen testing (via venous blood) yielded strongly positive results for eggs and wheat. A hyaluronic acid test and fecal occult blood test also yielded positive results. HBsAg, HBeAg, HBeAb, HBCAb, HCV, EBV-IgM tests; rheumatoid series; serum copper levels; coagulation; and routine urine analysis showed no abnormalities. Ultrasonography of the abdomen showed diffuse lesions and multiple solid nodules in the liver. Abdominal computed tomography showed hepatomegaly with small nodules under the right lobe of the liver and hypersplenotrophy with multiple nodules and enlarged splenic sinuses.

**Figure 1:**
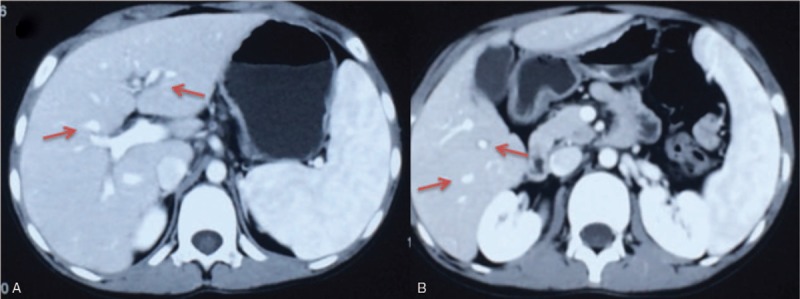
Hepatic computed tomography scan. Computed tomography scan shows a heterogeneous and nodular contour of the liver with mild splenomegaly.

In our hospital, physical examination was significant for purpura on both lower limbs and paroxysmal abdominal pain accompanied by hepatosplenomegaly with no Kayser–Fleischer rings detected in the eyes. The initial diagnosis was HSP, and more examinations were performed to explore the cause. The lab values were as follows: platelet count of 122 × 10^9^/L, with alanine aminotransferase level of 92.5 U/L and aspartate aminotransferase of 60.8 U/L. The patient also showed positivity for platelet glycoprotein antibody, CMV-DNA, EBV-DNA, and a rheumatoid series of antibodies, and a helicobacter pylori 13-C breath test was negative. An abdominal magnetic resonance imaging (MRI) showed hepatosplenomegaly. A computed tomography scan confirmed the heterogeneous and nodular contour of the liver with mild splenomegaly (Fig. 1).

**Figure 2:**
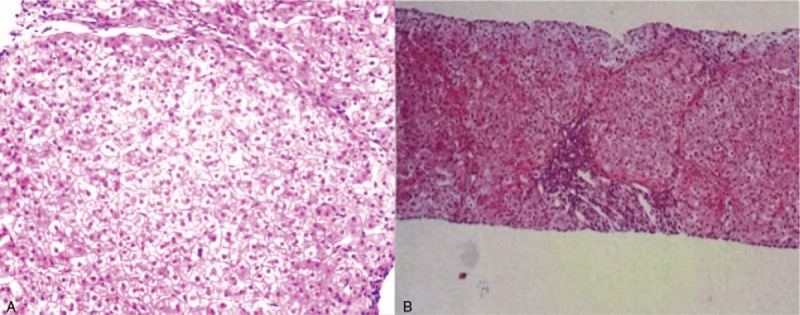
Pathological images from our hospital (A) and the other hospital (B). A, Extensive lobular formation in the liver tissue, vacuolar degeneration in liver cells, fibrous tissue hyperplasia in the portal area, infiltration with many lymphocytes and a small number of eosinophils and plasma cells. B, Final diagnosis (drug-induced liver injury, degree of the lesion: grade 2 sclerosis 4).

A needle biopsy of the liver was performed, which confirmed nodular cirrhosis and extensive lobular formations (Fig. 2A). To confirm the diagnosis, tissue was reviewed by NO. 302 Military Hospital of China, and a similar conclusion was drawn (drug-induced liver injury, degree of the lesion: grade 2 sclerosis 4) (Fig. 2B).

**Figure 3:**
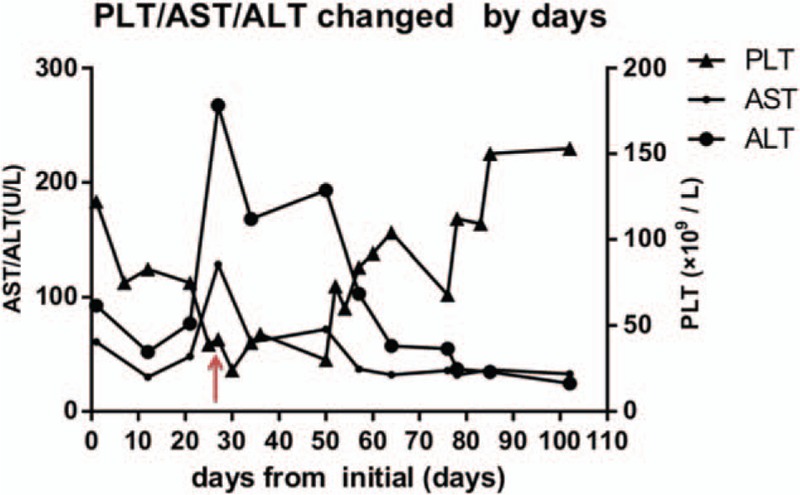
Change in PLT, AST, and ALT over days. The patient's platelet count gradually increased to above 150 × 109/L, and liver transaminase gradually decreased to a normal level. The arrow indicates the first day of the ucMSC injection. AST = aspartate amino transferase, alanine amino transferase, PLT = platelets.

With his history of drug abuse along with the pathology results, the child was diagnosed with nodular cirrhosis, with secondary allergic purpura and thrombocytopenia, etiologies related to drug use, and immune dysfunction. ucMSC transplantation was performed after written consent was obtained. ucMSCs (from Shandong cord blood bank) were transplanted 8 times/wk in 2 months, with a median cell count of 5.65 × 10^7^/L, which ranged from 5.48 to 5.98 × 10^7^/L. Methylprednisolone was tapered off after 1 month. As the skin rash almost resolved, the platelet count gradually increased (>150 × 10^9^/L), and liver transaminase gradually decreased to a normal level (Fig. 3). Abdominal ultrasound showed that the round nodules in the liver were smaller and fewer in number, and the spleen thickness was lesser (approximately 3.6 cm in contrast to 4.4 cm before). Follow-up assessments were performed after 3 and 6 months. During the follow-up visits, abdominal ultrasonography and abdominal MRI were performed. There was no presence of skin rash, and the patient had normal platelet count, had no nodules in the liver, and had a spleen thickness of 3 cm. The follow-up would be continued.

## 3 Discussion

The present case is the first report to show HSP with ITP. The reason for simultaneous existence of these 2 immune diseases is still uncertain.

Biopsy of hepatic tissue prompted a diagnosis of nodular cirrhosis (drug-induced). We consider that liver cirrhosis was caused by drug abuse for over 2 years, especially abuse of *Tripterygium wilfordii* (a traditional Chinese medicine). Thousands of drugs can cause liver damage including antibiotics, antituberculosis drugs, antifungal drugs, and Chinese herbal medicine.^[5,6]^ Considering the increased use of food additives and increased environmental pollution, drug-induced liver injury or hepatic failure has become increasingly common in clinical practice.^[5]^ Clinicians should pay more attention to drug-induced liver injury due to its seriousness and lack of specific manifestations. Monitoring liver function and avoiding medicating for an extended period of time is necessary.

As the largest reticuloendothelial cell phagocytosis system, the liver isolates and eliminates various types of external or endogenous antigens by phagocytosis.^[7]^ In this particular case, HSP accompanied by ITP may have resulted from the reduced ability of the liver to clear various antigens and a disorder of the immune system secondary to hepatic lesions. Thrombocytopenia may also have been related to splenomegaly and hypersplenism along with liver cirrhosis. Oral administration of *Tripterygium wilfordii* could also be directly related to liver damage. The time of liver cirrhosis occurrence was unclear due to the absence of abdominal ultrasound in early course of the disease. The diagnosis of thrombocytopenia was particularly unique in this case and has not been previously reported in other reports of children with HSP and liver cirrhosis.

In the absence of effective therapy, cirrhosis can lead to a series of complications such as venous hypertension, ascites, gastrointestinal bleeding, and hepatic encephalopathy. Stem cells with self-renewal ability and multidirectional differentiation potential can differentiate into a variety of cells with tissue regeneration and injury repair functions under certain conditions.^[3]^ A study found that stem cells transplanted into patients with liver cirrhosis could not only differentiate into liver cells in the liver-specific environment, but could also secrete some cytokines, leading to degradation of fibrous liver tissue and liver repair. MSC exist in a multitude of tissues, such as the bone marrow, umbilical cord blood, umbilical cord, and adipose tissue. Previous research has demonstrated that the 3 main mechanisms of MSC therapeutic effects are paracrine, cell replacement, and cell-to-cell contact.^[8]^ A previous study showed that hepatic stellate cells are the key mediators of liver fibrosis and play a crucial role in the pathogenesis of hepatic tissue fibrosis.^[9]^ Fibroblasts are derived from hepatocytes by the epithelial to mesenchymal transition and produce collagen.^[10–13]^ In the case of liver cirrhosis, transplanted human ucMSC could differentiate into hepatocyte-like cells, resulting in improved liver function.^[14]^ In the present case, the patient had improved liver function, resolution of allergic purpura, and a normal platelet count after ucMSC transplantation. Therefore, ucMSC can moderate the liver inflammatory response, reduce liver cell damage, and reduce the probability of hepatic failure. Previous work also showed that the therapeutic mechanism of ucMSC might be a paracrine mechanism.^[15]^ ucMSC therapy is approved in China for patients with liver cirrhosis. Further studies are required to confirm the therapeutic mechanism in vivo.

As a reliable therapy for many diseases, ucMSC transplantation represents a promising therapeutic strategy and area of research due to ucMSCs’ ability to differentiate and due to their higher proliferation potential and less severe immune reactions than conventional therapy.^[16–20]^ Due to the difficulty of clinical operation and high treatment cost, ucMSCs were infused via peripheral veins in this study.

This is a unique case of significant HSP with thrombocytopenia in a patient with under-lying liver cirrhosis. ucMSCs are a promising therapy for fibrotic liver disease. Although certain technical challenges exist and factors such as the occurrence of long-term adverse effects, injection rate and injection frequency, acceptable transplantation time window, and proper cell delivery require further studies, we believe that ucMSCs provide a novel approach for the treatment of liver cirrhosis.

## Acknowledgments

The authors thank NO. 302 Military Hospital of China for confirming the pathological diagnosis.

## Author contributions

**Conceptualization:** Kai Mu, Jing Zhang, Yan Gu, Hongjuan Li, Yan Han, Na Cheng, Xiaoyu Feng, Rongjun Zhang, Yuqi Zhao, Guoyu Ding, Hongmei Wang.

**Data curation:** Kai Mu, Yan Gu, Hongjuan Li, Xiaoyu Feng, Rongjun Zhang, Guoyu Ding, Hongmei Wang.

**Formal analysis:** Yan Gu, Hongjuan Li, Yan Han, Na Cheng, Xiaoyu Feng, Rongjun Zhang, Yuqi Zhao, Guoyu Ding.

**Investigation:** Jing Zhang, Hongmei Wang.

**Methodology:** Jing Zhang.

**Validation:** Yuqi Zhao.

**Visualization:** Jing Zhang.

**Writing – original draft:** Kai Mu, Hongmei Wang.

**Writing – review & editing:** Hongmei Wang.

## References

[1] Cogar BD Groshong TD Turpin BK Chylothorax in Henoch-Schonlein purpura: a case report and review of the literature. Pediatr Pulmonol 2005;39:563–7. 15830386 10.1002/ppul.20203

[2] Lu LL Liu YJ Yang SG Isolation and characterization of human umbilical cord mesenchymal stem cells with hematopoiesis-supportive function and other potentials. Haematologica 2006;91:1017–26. 16870554

[3] Zhang Z Lin H Shi M Human umbilical cord mesenchymal stem cells improve liver function and ascites in decompensated liver cirrhosis patients. J Gastroenterol Hepatol 2012;27:112–20. 22320928 10.1111/j.1440-1746.2011.07024.x

[4] Xue HL Zeng WZ Wu XL Clinical therapeutic effects of human umbilical cord-derived mesenchymal stem cells transplantation in the treatment of end-stage liver disease. Transplant Proc 2015;47:412–8. 25769583 10.1016/j.transproceed.2014.10.048

[5] Bjornsson E Review article: drug-induced liver injury in clinical practice. Aliment Pharmacol Ther 2010;32:3–13. 20374223 10.1111/j.1365-2036.2010.04320.x

[6] Ghabril M Fontana R Rockey D Drug-induced liver injury caused by intravenously administered medications: the Drug-induced Liver Injury Network experience. J Clin Gastroenterol 2013;47:553–8. 23388845 10.1097/MCG.0b013e318276bf00 PMC3681898

[7] Parker GA Picut CA Liver immunobiology. Toxicol Pathol 2005;33:52–62. 15805056 10.1080/01926230590522365

[8] Li T Xia M Gao Y Human umbilical cord mesenchymal stem cells: an overview of their potential in cell-based therapy. Expert Opin Biol Ther 2015;15:1293–306. 26067213 10.1517/14712598.2015.1051528

[9] Hernandez-Gea V Friedman SL Pathogenesis of liver fibrosis. Annu Rev Pathol 2011;6:425–56. 21073339 10.1146/annurev-pathol-011110-130246

[10] Iwano M Plieth D Danoff TM Evidence that fibroblasts derive from epithelium during tissue fibrosis. J Clin Invest 2002;110:341–50. 12163453 10.1172/JCI15518 PMC151091

[11] Puche JE Saiman Y Friedman SL Hepatic stellate cells and liver fibrosis. Compr Physiol 2013;3:1473–92. 24265236 10.1002/cphy.c120035

[12] Zeisberg M Yang C Martino M Fibroblasts derive from hepatocytes in liver fibrosis via epithelial to mesenchymal transition. J Biol Chem 2007;282:23337–47. 17562716 10.1074/jbc.M700194200

[13] Chen YL Lv J Ye XL Sorafenib inhibits transforming growth factor β1-mediated epithelial-mesenchymal transition and apoptosis in mouse hepatocytes. Hepatology 2011;53:1708–18. 21360571 10.1002/hep.24254

[14] Yang L Wang Y Wang X Effect of allogeneic umbilical cord mesenchymal stem cell transplantation in a rat model of hepatic cirrhosis. J Tradit Chin Med 2015;35:63–8. 25842730 10.1016/s0254-6272(15)30010-8

[15] Meirelles LS Fontes AM Covas DT Mechanisms involved in the therapeutic properties of mesenchymal stem cells. Cytokine Growth Factor Rev 2009;20:419–27. 19926330 10.1016/j.cytogfr.2009.10.002

[16] Pittenger MF Mackay AM Beck SC Multilineage potential of adult human mesenchymal stem cells. Science 1999;284:143–7. 10102814 10.1126/science.284.5411.143

[17] Mareschi K Biasin E Piacibello W Isolation of human mesenchymal stem cells: bone marrow versus umbilical cord blood. Haematologica 2001;86:1099–100. 11602418

[18] Togel F Weiss K Yang Y Vasculotropic, paracrine actions of infused mesenchymal stem cells are important to the recovery from acute kidney injury. Am J Physiol Renal Physiol 2007;292:F1626–35. 17213465 10.1152/ajprenal.00339.2006

[19] Yan Y Xu W Qian H Mesenchymal stem cells from human umbilical cords ameliorate mouse hepatic injury in vivo. Liver Int 2010;29:356–65. 10.1111/j.1478-3231.2008.01855.x 19141029

[20] Xu H Qian H Zhu W Mesenchymal stem cells relieve fibrosis of Schistosoma japonicum-induced mouse liver injury. Exp Biol Med (Maywood) 2012;237:585–92. 22678013 10.1258/ebm.2012.011362

